# Dietary management experiences and support needs of primary caregivers of children with asthma and food allergy: a qualitative descriptive study

**DOI:** 10.3389/fnut.2026.1904972

**Published:** 2026-08-12

**Authors:** Kejimu Sunzi, Quanmin Deng, Qin Huang, Yiluo Xu, Ting Zeng, Hui Luo, Cheng Lei

**Affiliations:** 1 Department of Nursing, Deyang People's Hospital, Deyang, China; 2 Department of Pediatrics, Women and Children's Center, Deyang People's Hospital, Deyang, China; 3 Department of Nursing, Sichuan Nursing Vocational College, Deyang, China; 4 Department of Nursing, Sichuan Clinical Research Center for Cancer, Sichuan Cancer Hospital & Institute, Sichuan Cancer Center, University of Electronic Science and Technology of China, Chengdu, China

**Keywords:** caregivers, childhood asthma, dietary management, food allergy, nutrition counseling, qualitative research, support needs, thematic analysis

## Abstract

**Objective:**

Children living with both asthma and food allergy require daily management that connects respiratory symptoms, allergen avoidance, emergency preparedness, and adequate nutrition. This study aimed to explore dietary management experiences and support needs among primary caregivers of school-age children with physician-diagnosed asthma and food allergy in Deyang, China.

**Methods:**

A qualitative descriptive design was used. Semi-structured Mandarin interviews were conducted with 24 primary caregivers, and the data were analyzed using reflexive thematic analysis.

**Results:**

Four themes were developed: (1) managing “double danger” when food allergy reactions, asthma symptoms, respiratory infections, and culturally mediated food concerns overlapped; (2) building protective food routines at home, school, and social events; (3) balancing safety, nutrition, and normal childhood; and (4) needing integrated, practical, and credible support across asthma, food allergy, nutrition, school communication, and emergency planning.

**Conclusion:**

Caregivers' dietary work extends beyond allergen avoidance and involves ongoing risk interpretation, emotional labor, and coordination across settings. The findings support the need for clearer, family-centered counseling and school-facing tools, but do not establish the effectiveness of any specific intervention. Future intervention development should test integrated education, individualized dietary planning, and age-appropriate self-management support with caregivers, children, clinicians, dietitians, and schools.

## 1 Introduction

Asthma is one of the most common chronic diseases in childhood and remains a major contributor to preventable morbidity, impaired quality of life, school absenteeism, and family burden. The Global Initiative for Asthma (GINA) continues to emphasize that effective asthma care requires sustained symptom monitoring, medication adherence, trigger awareness, and an individualized action plan (1). In China, childhood asthma has become an increasingly visible public health concern. National epidemiological surveys reported that asthma prevalence among children aged 0–14 years increased from 0.98% in 1990 to 1.97% in 2000 and 3.02% in 2010 (2). A more recent Global Burden of Disease 2021 analysis estimated 2.26 million new childhood asthma cases in China in 2021 and showed that children aged 5–9 years had the largest number of prevalent cases (3). These data underscore the importance of family-centered asthma management during school age, when children begin to eat, learn, and socialize in settings beyond direct parental supervision.

Food allergy is likewise an increasingly recognized chronic pediatric condition that can affect nutrition, eating routines, social participation, and caregiver anxiety. International prevalence estimates vary by case definition, diagnostic confirmation, age, and region, but population-based data consistently show that food allergy affects a meaningful proportion of children (4, 5). In the United States, a large national survey estimated that 7.6% of children had convincing parent-reported food allergy, and 40% of food-allergic children had multiple food allergies (6). In China, a population-based survey of 8,856 primary school children aged 6–11 years in Jiangxi Province reported parent-reported food allergy and doctor-diagnosed food allergy prevalence rates of 6.2% and 3.3%, respectively; shrimp, mango, and mollusks were the leading reported allergenic foods, and 45.32% of children with parent-reported food allergy experienced severe allergic reactions (7). A recent review of pediatric food allergy in China further highlighted regional variation in allergen profiles, diagnostic heterogeneity, limited availability of allergy specialists, and uneven access to epinephrine autoinjectors (8).

Asthma and food allergy commonly coexist, and children with food allergy are more likely to have asthma than children without food allergy (9, 10). Asthma is also an important risk factor for severe or fatal food-related anaphylaxis, particularly when asthma is poorly controlled (5, 9, 11). In daily life, this comorbidity complicates symptom interpretation. Coughing, wheezing, throat discomfort, rash, vomiting, or breathlessness after eating may suggest an allergic reaction, asthma exacerbation, viral infection, reflux, choking, anxiety, or an ordinary childhood symptom. Dietary management may therefore become both a safety practice and a source of uncertainty.

Clinical guidance emphasizes confirmation of food allergy diagnosis, individualized allergen avoidance, dietary advice, written treatment plans, and education on recognizing and treating allergic symptoms (1, 5, 11). Recent food allergy guidance highlights the importance of dietary counseling, emergency planning, and psychosocial support for patients and families living with IgE-mediated food allergy (5, 11). Nutrition-focused reviews also caution that avoidance diets, although necessary for confirmed allergens, may compromise dietary variety, growth, and micronutrient adequacy if not accompanied by appropriate substitution and follow-up (12, 13). For children, these recommendations must be translated into ordinary routines: buying groceries, reading labels, preparing school lunches, refusing unsafe foods, negotiating family meals, and responding to uncertain symptoms.

The existing qualitative literature suggests that caregivers' beliefs about food can shape childhood asthma management. In a Singaporean focus group study, caregivers of children with asthma perceived infection, food, and exercise as important asthma-related concerns; some families restricted certain foods because they believed these foods could trigger asthma (14). Separately, qualitative studies of families managing pediatric food allergy describe anxiety, loss of normalcy, strained relationships, financial burden, daily vigilance, and a need for credible, accessible resources (15, 16). Studies among adolescents with food allergy and their caregivers further show that families must prepare safe foods, advocate in social settings, carry emergency medication, and gradually transfer self-management responsibility to the child (17). Research on caregiver burden in severe pediatric asthma and systematic reviews of food allergy-related quality of life also indicate that chronic allergic disease management can disrupt family routines, heighten emotional distress, and increase support needs (18, 19).

In the Chinese context, dietary decisions may also be shaped by culturally embedded concepts such as foods perceived as “heaty,” “cold,” or “phlegm-producing.” These concepts should not be treated only as misinformation; they are part of the social language through which families interpret symptoms, negotiate intergenerational advice, and decide when medical advice fits ordinary meals.

However, these strands of literature often remain separate. Studies of childhood asthma may mention food-related beliefs without focusing on clinically confirmed food allergy, while studies of food allergy may include children with asthma but do not necessarily analyze how comorbid asthma changes dietary management. This leaves a practical gap for clinicians: families may receive asthma education in respiratory clinics, food avoidance advice in allergy clinics, and nutrition advice only when growth or dietary adequacy becomes a concern. Caregivers may be left to integrate these messages on their own.

This qualitative descriptive study explored the dietary management experiences and support needs of primary caregivers of school-age children with both asthma and food allergy. We focused on school-age children because this developmental period involves increasing independence, school meals, peer interactions, and early self-management learning while caregivers remain centrally responsible for safety and nutrition.

## 2 Materials and methods

### 2.1 Study design

We used a qualitative descriptive design with reflexive thematic analysis. Reporting was guided by the Standards for Reporting Qualitative Research and the Consolidated Criteria for Reporting Qualitative Research for interview studies (20, 21). The design and analytic approach were informed by established literature on thematic analysis, qualitative trustworthiness, and qualitative description (22–25).

A completed COREQ checklist was prepared as Supplementary material to show where each reporting item is addressed in the manuscript and Supplementary Tables.

### 2.2 Setting

The study was conducted in the pediatric respiratory and allergy outpatient clinics of the Women and Children's Center, Deyang People's Hospital, Deyang, China. This tertiary hospital-based pediatric service provides specialist care for children with asthma, allergic rhinitis, eczema, food allergy, and complex atopic comorbidities. Interviews were conducted between October 2025 and March 2026 in a private consultation room or by encrypted video call, according to caregiver preference.

### 2.3 Participants and sampling

Primary caregivers were eligible if they were aged 18 years or older; cared for a child aged 6–12 years; had primary responsibility for the child's daily diet; and could participate in a Mandarin interview. Eligible children were required to have both physician-diagnosed asthma documented in the medical record and physician-diagnosed food allergy. Asthma diagnosis was based on the treating physician's clinical diagnosis recorded in the child's medical file, including recurrent respiratory symptoms and clinical assessment consistent with pediatric asthma practice. Asthma control at recruitment was abstracted from the medical record and categorized as controlled, partly controlled, or uncontrolled according to symptom frequency, night waking, activity limitation, reliever use, and recent exacerbation history as documented by the clinical team. Food allergy was defined as a physician diagnosis based on clinical history plus supporting allergy testing and/or oral food challenge where available. Food allergy phenotype was recorded as IgE-mediated, non-IgE-mediated, mixed, or not specified when this information was available in the clinical record. Severe systemic allergic reaction was defined as a reaction involving respiratory or cardiovascular symptoms, two or more organ systems, or emergency department treatment. Caregivers whose child had asthma-related or culturally mediated food beliefs without physician-diagnosed food allergy were not eligible.

Asthma-control status was not determined using a study-administered standardized questionnaire such as the Childhood Asthma Control Test. It was abstracted from the treating clinician's assessment in the medical record, using GINA-aligned clinical domains; this reliance on routine documentation is acknowledged as a limitation. Food-allergy phenotype and diagnostic evidence were also abstracted from clinical records. Because oral food challenge was available for only six children, diagnostic certainty was heterogeneous. The study term “severe systemic allergic reaction” was a pragmatic operational category rather than a retrospective formal diagnosis of anaphylaxis; reaction history was obtained from caregiver report and checked against the medical record where documentation was available.

Caregivers were excluded if the child had a chronic gastrointestinal, metabolic, renal, endocrine, or developmental condition requiring a therapeutic diet unrelated to food allergy, or if the caregiver was unable to provide informed consent.

Purposive sampling was used to maximize variation in child age, sex, asthma control, type and number of food allergens, history of severe allergic reaction, caregiver relationship to the child, education level, and urban or suburban residence.

Recruitment was tracked prospectively. Of 41 caregivers approached, 36 were screened; five were not screened because they could not be contacted after invitation (*n* = 3) or had no time for screening (*n* = 2). Eight screened caregivers were ineligible because the child had no confirmed food allergy (*n* = 5), was outside the 6–12-year age range (*n* = 2), or required a therapeutic diet unrelated to food allergy (*n* = 1). Of 28 eligible caregivers, 24 enrolled and four declined because of time constraints (*n* = 2), concern about audio recording (*n* = 1), or perceiving the topic as stressful (*n* = 1); no participant withdrew. Seventeen interviews were conducted in clinic and seven by video. Recruitment was considered complete using information power (26): the study aim was focused, the sample was specific to children with comorbid asthma and food allergy, interviews generated detailed accounts of daily dietary management, and interviews 21–24 yielded no new themes while confirming variation across clinical profiles.

**Figure 1:**
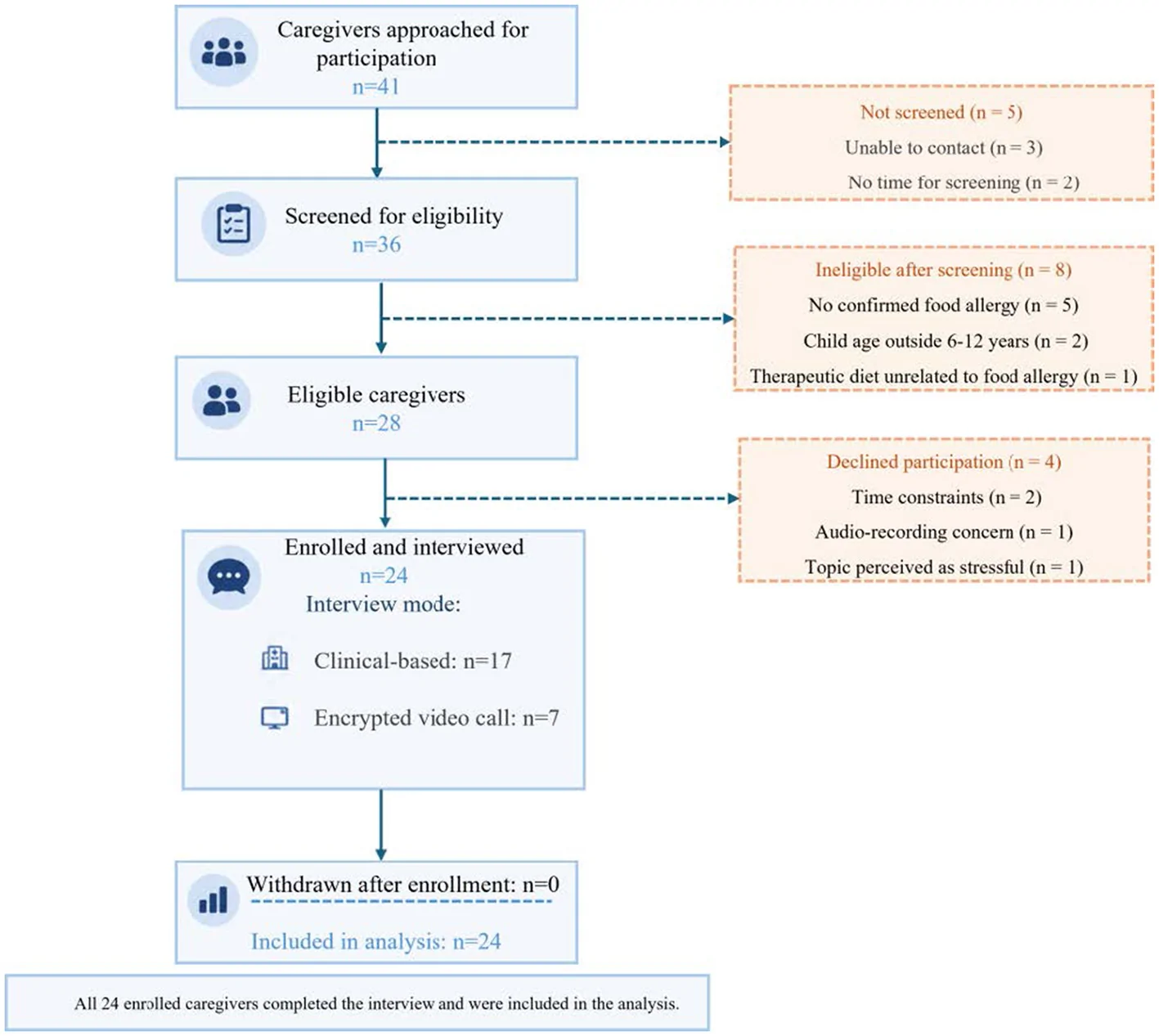
Recruitment flow diagram.

Analytic sufficiency was reviewed during team meetings as interviews accumulated. By interviews 21–24, no new dimensions were being added to the four developing themes, although later interviews continued to elaborate contrasts by asthma control, number of food allergies, severe-reaction history, and dietitian exposure. Fathers (*n* = 4), grandmothers (*n* = 2), children with non-IgE-mediated allergy (*n* = 2), and families with ongoing dietitian follow-up (*n* = 2) were comparatively less represented; therefore, the analysis does not claim subgroup-specific saturation or population-level differences (Figure 1).

### 2.4 Data collection

Semi-structured interviews were conducted by CL, who was trained in qualitative interviewing and was not involved in the children's direct clinical care. The interview guide was developed through discussion among pediatric respiratory clinicians, allergy specialists, clinical dietitians, and qualitative researchers. Two pilot interviews were conducted to refine wording and sequencing; because no substantial changes were made, pilot interviews were retained in the dataset.

CL is a registered nurse with a PhD in Epidemiology and Health Statistics, several years of clinical experience, and research expertise in study methodology, clinical care, and digital health interventions. At the time of the study, CL was a postdoctoral researcher at Sichuan Cancer Hospital and was independent of the pediatric respiratory and allergy clinics involved in participant recruitment. CL was not involved in the children's clinical care and had no prior clinical relationship with the participating caregivers. This combined clinical and methodological expertise informed CL's role in conducting the interviews, providing guidance on qualitative methods, and safeguarding the rigor and integrity of the research process.

The interview guide included open-ended questions about diagnosis experiences, perceived links between food and asthma, daily allergen avoidance, meal preparation, school and social eating, nutrition concerns, family decision-making, emergency preparedness, experiences with healthcare information, and desired support. Example questions included: “Can you tell me about a recent situation when you had to make a dietary decision because of your child's asthma or food allergy?”; “How do you decide whether a symptom is related to food allergy, asthma, infection, or something else?”; “What is most difficult about managing your child's diet at school or outside the home?”; and “What kind of support from healthcare professionals would make daily management easier?”

Interview duration, mode, and completion status were recorded for each participant and summarized descriptively. Interviews were audio-recorded with permission, transcribed verbatim, and anonymized. Field notes were written within 24 h of each interview to capture interview context, non-verbal observations, and early analytic impressions. Participants were assigned anonymous codes.

The overall median interview duration was 42 min (range, 30–60 min). The median duration was 40 min for clinic-based interviews (range, 35–60 min) and 40 min for video interviews (range, 30–50 min). No interviews were unusually short or incomplete.

Interviews were conducted and transcribed in Mandarin. Coding and theme development were conducted primarily in Mandarin to preserve participants' meanings. English quotations were translated after theme development, consistent with recommendations for documenting translation decisions and preserving cultural meaning in cross-language qualitative research (27). Quotations were translated by a bilingual nursing researcher, checked against the Mandarin transcripts by a second bilingual team member, and reviewed by team members familiar with pediatric asthma, food allergy, and Chinese dietary language. Culturally specific terms such as “heaty,” “cold foods,” and “phlegm-producing” were retained with concise explanatory wording rather than replaced by biomedical terms. Six participants reviewed a brief thematic summary, rather than their individual transcripts, and commented on whether the themes were recognizable.

### 2.5 Research team and reflexivity

The research team included pediatric respiratory nurses, a pediatric allergist, a clinical dietitian, and a qualitative health researcher. CL was a qualitative health researcher with pediatric nursing research experience and was not involved in the children's direct clinical care. Team members entered the study with clinical experience suggesting that some caregivers either over-restrict foods because of fear or under-recognize food-allergy risk in social settings. To reduce premature interpretation, the interviewer used open-ended prompts and avoided correcting caregivers during interviews unless a safety concern required clinical referral after the interview. Analytic memos documented assumptions about diet, allergy risk, asthma control, family responsibility, and culturally embedded food beliefs. Regular team meetings were used to compare interpretations across clinical and qualitative perspectives.

CL's position as an external interviewer reduced the likelihood that participants would perceive the interview as part of their child's clinical care, but did not eliminate positional influence. Reflexive memos considered how CL's nursing background, clinical experience, methodological orientation, and distance from the pediatric service could shape questioning and interpretation. During team discussions, clinical assumptions about “appropriate” avoidance, asthma control, family responsibility, and culturally mediated food beliefs were treated as analytic positions to be examined rather than as standards against which caregiver accounts were judged.

### 2.6 Data analysis

Data were analyzed using reflexive thematic analysis (22, 28, 29). First, two researchers read all Mandarin transcripts to become familiar with the dataset and wrote analytic memos. Second, initial coding was conducted in Mandarin; transcripts, codes, and memos were organized using NVivo 14, and 126 initial codes were generated. Third, codes were reviewed and grouped into candidate categories reflecting caregivers' risk interpretation, avoidance practices, nutritional concerns, school and social negotiation, and support needs. Fourth, candidate themes were compared across caregiver relationship, child age group, asthma control status, number of food allergens, history of severe systemic reaction, and previous dietitian consultation. Fifth, candidate themes were reviewed against the full dataset, including negative and divergent cases such as families reporting adequate school support, effective dietary routines, or less anxiety after dietitian consultation. Finally, themes were defined and named around the central phenomenon of dietary management under comorbid asthma and food allergy.

Inter-coder reliability was not calculated because the analysis followed an interpretive reflexive thematic approach rather than a coding-reliability design. Differences in interpretation were handled through discussion, memo review, and return to transcript context. These discussions were used to refine theme boundaries rather than to force coding consensus. An audit trail documented recruitment decisions, interview guide refinements, coding decisions, memo development, theme revisions, and quotation selection.

Coding was collaborative and iterative rather than two independent coding streams designed to achieve agreement. No fixed codebook was imposed in advance; working codes were revised, combined, and discarded as interpretation developed. Reflexive memos were used to identify how professional assumptions influenced code labels and candidate themes. Differences were explored by returning to transcript context and considering alternative readings; discussion aimed to deepen interpretation and define theme boundaries, not to calculate agreement or force consensus.

Quantitative information was used only to describe the sample and interview process. Categorical variables, including caregiver relationship, child sex, child age group, asthma control status, food allergy profile, history of severe systemic allergic reaction, written emergency plan, previous dietitian consultation, and residence, were summarized as frequencies and percentages. No inferential statistical testing was performed because the study was designed for qualitative depth and transferability rather than estimation of population-level associations.

### 2.7 Trustworthiness

Credibility was supported through purposive sampling, in-depth interviews, iterative data collection, peer debriefing, comparison of cases with different asthma control and allergy profiles, and checking translated quotations against the original Mandarin transcripts. Dependability was supported through an audit trail documenting recruitment, interview-guide changes, coding decisions, memo development, and theme revision. Confirmability was strengthened through reflexive memoing and discussion of alternative interpretations. Transferability was supported by detailed description of the clinical setting, participants, family management context, and cultural features of food-related interpretation (23, 24). A brief summary of preliminary findings was shared with six caregivers for member reflection; they reported that the themes were recognizable and suggested greater emphasis on school-meal communication.

### 2.8 Ethical considerations

The study protocol was approved by the Deyang City People's Hospital Ethics Committee (2025-04-043-K01). Written informed consent was obtained from all participating caregivers before the interviews, including permission to access, extract, and use relevant clinical information from the child's medical record for research purposes. Age-appropriate assent was also obtained from participating children in accordance with the approved study protocol. Participants were informed that participation was voluntary, that refusal or withdrawal would not affect the child's medical care, and that de-identified quotations could be used in publications. Audio recordings, transcripts, and extracted clinical data were stored on password-protected institutional servers accessible only to authorized members of the research team.

## 3 Results

### 3.1 Participant characteristics

Overall, 24 primary caregivers participated in the study. Table 1 presents clinical, caregiver, dietary, household, and school-context characteristics relevant to interpretation of dietary management experiences.

**Table 1:** Participant characteristics (*N* = 24).

Characteristic	*n* (%) or summary
Caregiver relationship: mother	18 (75.0)
Caregiver relationship: father	4 (16.7)
Caregiver relationship: grandmother	2 (8.3)
Caregiver age, years	36.8 +/- 5.9; range 27–61
Caregiver education: junior high school or below	5 (20.8)
Caregiver education: senior high/vocational school	7 (29.2)
Caregiver education: college or above	12 (50.0)
Caregiver employment: full-time	10 (41.7)
Caregiver employment: part-time/self-employed	6 (25.0)
Caregiver employment: homemaker	6 (25.0)
Caregiver employment: retired grandparent	2 (8.3)
Monthly household income: < 5,000 CNY	6 (25.0)
Monthly household income: 5,000–9,999 CNY	11 (45.8)
Monthly household income: >=10,000 CNY	7 (29.2)
Household structure: nuclear family	15 (62.5)
Household structure: three-generation household	9 (37.5)
Child sex: male	15 (62.5)
Child sex: female	9 (37.5)
Child age 6–8 years	10 (41.7)
Child age 9–12 years	14 (58.3)
Asthma controlled	8 (33.3)
Asthma partly controlled	9 (37.5)
Asthma uncontrolled	7 (29.2)
Asthma medication: inhaled corticosteroid-containing controller	17 (70.8)
Asthma medication: reliever inhaler available	20 (83.3)
Asthma medication: leukotriene receptor antagonist	10 (41.7)
Time since asthma diagnosis, years	3.1 (IQR 1.8–5.0)
Single food allergy	9 (37.5)
Multiple food allergies	15 (62.5)
Specific food allergens: egg	9 (37.5)
Specific food allergens: cow's milk	8 (33.3)
Specific food allergens: shrimp/mollusks	7 (29.2)
Specific food allergens: peanut/tree nuts	6 (25.0)
Specific food allergens: mango/other fruit	4 (16.7)
Specific food allergens: wheat	3 (12.5)
Specific food allergens: soy	2 (8.3)
Food allergy phenotype: IgE-mediated	18 (75.0)
Food allergy phenotype: non-IgE-mediated	2 (8.3)
Food allergy phenotype: mixed	3 (12.5)
Food allergy phenotype: not specified in record	1 (4.2)
Food allergy diagnostic evidence: clinical history	24 (100.0)
Food allergy diagnostic evidence: serum specific IgE	18 (75.0)
Food allergy diagnostic evidence: skin prick testing	15 (62.5)
Food allergy diagnostic evidence: oral food challenge	6 (25.0)
Number of avoided foods: 1–2	9 (37.5)
Number of avoided foods: 3–5	11 (45.8)
Number of avoided foods: >=6	4 (16.7)
Reaction history: cutaneous symptoms	17 (70.8)
Reaction history: gastrointestinal symptoms	10 (41.7)
Reaction history: respiratory symptoms	8 (33.3)
History of severe systemic allergic reaction	9 (37.5)
Epinephrine autoinjector available	5 (20.8)
Written emergency plan available	13 (54.2)
Any previous dietitian consultation	7 (29.2)
Dietitian consultation: one-off	5 (20.8)
Dietitian consultation: ongoing/follow-up	2 (8.3)
Previous nutritional assessment	6 (25.0)
Caregiver-reported growth or nutrition concern	11 (45.8)
School eating: home-packed meals only	10 (41.7)
School eating: school meals with restrictions	9 (37.5)
School eating: mixed school meals and home-packed food	5 (20.8)
Urban residence	16 (66.7)
Suburban residence	8 (33.3)

Values are *n* (%) unless otherwise stated. Categories for asthma medication, specific food allergens, diagnostic evidence, and reaction history are not mutually exclusive; therefore, percentages may exceed 100%. Caregiver, household, school-eating, avoided-food, nutrition-concern, emergency-preparedness, and dietitian-use variables were caregiver-reported. Asthma diagnosis/control, prescribed medication, food-allergy phenotype, diagnostic evidence, and documented reaction history were abstracted from medical records; caregiver-reported reaction details were checked against records where available.

Four themes were developed: (1) managing “double danger”; (2) building protective food routines; (3) balancing safety, nutrition, and normal childhood; and (4) needing integrated, practical, and credible support (Table 2).

**Table 2:** Themes, subthemes, and practice implications.

Theme	Analytic focus and variation	Illustrative examples	Practice implications
Managing “double danger”	Caregivers distinguished confirmed allergens from suspected asthma-related food triggers; uncertainty was greater when asthma was uncontrolled or prior systemic reactions had occurred.	C04: peanut was clear, but cough after ice cream created uncertainty; C02: throat symptoms plus cough caused fear of choosing the wrong first treatment.	Provide integrated education on symptom recognition and how asthma and allergy action steps differ.
Building protective food routines	Families used label reading, separate cooking, school negotiation, and social eating preparation; divergent cases included schools that allowed home-packed meals or medication storage.	C06: packed lunch plus backup snacks; C13: teacher kindness but limited capacity to monitor every bite.	Offer practical tools for home, school, restaurants, travel, and relatives.
Balancing safety, nutrition, and normal childhood	Multiple allergies and avoidance of staple or protein-rich foods intensified nutrition concerns; dietitian consultation appeared to reduce uncertainty for some families.	C09: concern about egg and milk avoidance; C18: child distress when excluded from birthday cake; C23: prolonged over-restriction after an emergency visit.	Include dietitians and developmentally appropriate self-management support.
Needing integrated, practical, and credible support	Caregivers wanted one coherent plan rather than separate asthma, allergy, and nutrition messages; needs included digital resources, school letters, and emotional reassurance.	C07: wanted one paper distinguishing forbidden foods, safe foods, allergy symptoms, asthma symptoms, and first steps; C12: wanted menus and substitutions.	Develop multidisciplinary, family-centered, and school-facing support that is tested in future intervention research.

### 3.2 Theme 1: managing “double danger”

Caregivers described dietary management as complicated by the coexistence of asthma and food allergy. They did not simply avoid confirmed allergens; they also interpreted respiratory symptoms, skin changes, gastrointestinal discomfort, and viral illness through a lens of overlapping danger. A cough after eating could be read as early asthma, a mild allergic reaction, choking, reflux, or infection. This uncertainty shaped how quickly caregivers sought medical help, whether they removed foods from the child's diet, and how confident they felt in daily decisions.

Several caregivers distinguished “known allergens” from “suspected triggers.” Known allergens were foods that had caused immediate reactions or were confirmed by clinicians. Suspected triggers were broader and often included cold drinks, spicy foods, seafood, sweets, processed snacks, or foods described as “heaty” or “phlegm-producing.” Caregivers used family experience, online information, traditional dietary beliefs, and clinician advice to interpret these foods.

“Peanut is clear. We know he cannot eat it. But when he coughs after ice cream, I do not know whether it is asthma, cold food, or just coincidence. So slowly we stopped many things, not only peanut.” (C04, mother of a 7-year-old boy)

“The allergy doctor told us milk was not the problem, but my mother-in-law still thinks milk makes phlegm. When he wheezes, everyone asks what he ate, not whether he used the inhaler correctly.” (C11, mother of a 9-year-old boy)

The “double danger” was most intense among caregivers whose children had previous severe allergic reactions. For these caregivers, respiratory symptoms during an allergic reaction were especially frightening because wheeze and breathlessness were already associated with asthma attacks.

“When she says her throat feels strange, I immediately think of allergy. If she coughs at the same time, I panic because asthma is also there. I am afraid I will choose the wrong medicine first.” (C02, mother of a 10-year-old girl)

Some caregivers reported that different clinicians emphasized different aspects of care. Respiratory clinicians focused on inhaled medications and asthma control; allergy clinicians emphasized allergen avoidance and emergency response; nutrition guidance was often received only if caregivers asked about substitutes. Families were left to integrate the messages.

“In the asthma clinic they ask about coughing and inhalers. In the allergy clinic they ask about peanuts and eggs. But at home it is one child, one meal, one night of coughing. I need someone to put the whole picture together.” (C17, father of an 8-year-old boy)

Variation across cases suggested that caregivers of children with uncontrolled or partly controlled asthma described greater ambiguity when respiratory symptoms occurred after eating, whereas caregivers of children with controlled asthma more often framed uncertainty around school and social exposure. Families with multiple food allergies also described broader interpretive work because the boundary between confirmed allergens, suspected triggers, and culturally avoided foods was harder to maintain. These culturally embedded food categories operated as family explanatory models and negotiation tools, not simply as incorrect biomedical beliefs.

Across these comparisons, uncertainty was not uniform. Caregivers of children with controlled asthma more often described familiar respiratory routines and focused on exposure outside the home, whereas uncontrolled asthma and multiple allergies widened the range of symptoms and foods that required interpretation. The accounts did not support treating emergency-plan availability, epinephrine-autoinjector availability, or school-meal type as determinants of confidence; these characteristics were used as contextual sensitizing information rather than variables for association testing.

Across the dataset, culturally mediated food categories interacted with clinical advice rather than operating as a separate belief system. They shaped how cough or wheeze was attributed, how grandparents and parents negotiated food reintroduction, and whether reassurance that a food was non-allergenic was trusted. The accounts suggest that specific explanations linking symptoms, confirmed allergens, and asthma-management steps were easier for caregivers to use in family negotiations than brief instructions simply to avoid or permit a food.

### 3.3 Theme 2: building protective food routines

Caregivers described extensive practical work to create safe eating routines. At home, this included reading ingredient lists, checking manufacturing warnings, separating utensils, cooking different meals for the child, and training grandparents or domestic helpers. Outside the home, families prepared safe snacks, contacted teachers, avoided shared foods, and sometimes declined birthday parties or school activities involving food.

“Every morning I pack two boxes: one lunch and one backup. If the school has a special activity, I pack another snack because I am afraid someone will give him cake or candy.” (C06, mother of a 6-year-old boy)

Label reading was both a safety strategy and a source of anxiety. Caregivers found allergen labels inconsistent, difficult to interpret, or absent in small bakeries and restaurants. Families with multiple allergies reported that the number of excluded foods made ordinary shopping time-consuming and expensive.

“The package says ‘may contain nuts.' Does that mean definitely unsafe or just the factory warning? I stand in the supermarket comparing labels for a long time. Sometimes I just give up and buy the same food again.” (C21, mother of an 11-year-old girl)

School was a major site of negotiation. Caregivers wanted children to participate in meals and activities but worried about food sharing, teacher awareness, and emergency preparedness. Some schools allowed home-packed meals and kept medication in the nurse's office; others had limited protocols.

“The teacher is kind, but there are more than forty children. I cannot expect her to watch every bite. My son knows not to share food, but children forget when they are playing.” (C13, father of a 9-year-old boy)

Social eating required anticipatory planning. Caregivers contacted restaurant staff, brought food from home, or ate before attending events. Some families avoided restaurants entirely after experiencing ambiguous or dismissive responses from food service staff.

“When I ask whether there is egg in the sauce, some people say, 'A little should be fine.' For us, a little is not fine. After hearing that, I prefer to bring food myself.” (C01, mother of a 7-year-old girl)

Not all cases followed a single narrative of inadequate support. Several caregivers described workable arrangements when teachers accepted home-packed meals, medication storage was permitted, or school staff were willing to contact caregivers before food-related activities. These divergent cases suggested that the burden of dietary management was reduced when schools had clear communication channels and when caregivers trusted that adults outside the home understood the child's specific confirmed allergens.

### 3.4 Theme 3: balancing safety, nutrition, and normal childhood

Although caregivers prioritized safety, many worried that the child's diet had become too narrow. This was especially prominent in families avoiding milk, egg, wheat, or multiple protein sources. Caregivers worried about calcium intake, growth, appetite, and the psychological effects of repeated restriction. Only seven caregivers had received dietitian counseling, and several relied on internet searches or parent groups for substitutions.

“He cannot eat egg and milk. I know there are substitutes, but I am not sure whether the nutrition is enough. He is thinner than classmates, so I always wonder whether my caution is harming him in another way.” (C09, mother of an 8-year-old boy)

Caregivers also described tension between safety and normal childhood. Some children felt embarrassed eating separate food, refusing snacks, or being watched closely. Older children wanted more independence but caregivers were uncertain when to trust them.

“She says, ‘Why am I different again?' I can prepare safe food, but I cannot prepare her feelings when everyone else eats the birthday cake.” (C18, mother of a 10-year-old girl)

Family disagreement complicated dietary decisions. Grandparents sometimes believed that strict avoidance would make the child “weaker” or that small exposures could build tolerance. Other relatives believed the child should avoid many additional foods because of asthma, even when these foods were not confirmed allergens. Caregivers often acted as mediators between medical advice and family beliefs.

“My father says children should not be protected too much, just try a little and see. My husband says avoid everything to be safe. I am in the middle, trying to follow the doctor and keep peace at dinner.” (C15, mother of a 12-year-old boy)

Several caregivers described “dietary narrowing” after frightening events. A severe allergic reaction or asthma hospitalization made families remove more foods than medically indicated. These restrictions sometimes persisted for months because caregivers lacked confidence to reintroduce non-allergenic foods.

“After the emergency visit, I stopped seafood, nuts, mango, chocolate, cold drinks, and many snacks. Later the doctor said only shrimp was confirmed, but I was afraid to add things back.” (C23, grandmother of a 6-year-old boy)

Comparisons across cases suggested that families managing multiple allergies, staple-food avoidance, or previous systemic reactions described the strongest tension between safety and dietary adequacy. In contrast, caregivers who had received dietitian consultation more often described concrete substitution strategies and greater confidence distinguishing medically necessary avoidance from broader precautionary restriction. Mothers frequently described the day-to-day planning burden, fathers more often described school or restaurant negotiation, and grandparents' accounts highlighted intergenerational disagreement about strengthening the child through exposure or avoiding foods considered harmful for asthma.

Some families described relatively stable routines rather than persistent high anxiety. This was most evident when confirmed allergens were clearly defined, school communication was workable, children could follow simple refusal and food-sharing rules, or dietitian advice provided concrete substitutions. These cases did not remove vigilance but showed that burden and uncertainty varied across contexts.

### 3.5 Theme 4: needing integrated, practical, and credible support

Caregivers wanted support that was integrated across asthma, food allergy, and nutrition. They did not want only general education about “healthy eating” or only a list of foods to avoid. They wanted individualized explanations of confirmed allergens, suspected but unconfirmed foods, safe alternatives, symptom recognition, emergency response, and how asthma control affected the family's confidence in managing allergic reactions.

“I need one paper that says: these foods are truly forbidden, these are okay, these symptoms mean allergy, these symptoms mean asthma, and this is what to do first.” (C07, mother of an 8-year-old girl)

Caregivers preferred practical tools: sample meal plans, safe snack lists, label-reading examples, school letters, restaurant communication cards, and action plans that teachers and relatives could understand. Several requested a joint clinic or education session involving a respiratory nurse, allergist, and dietitian.

“The doctor tells us the principle, but at home I need the menu. What breakfast? What calcium? What can he bring to school? I hope the nutrition teacher can work with the asthma and allergy doctors.” (C12, mother of a 9-year-old boy)

Digital information was a double-edged resource. Caregivers searched online frequently, but contradictory advice increased anxiety. Parent groups provided emotional support and practical tips but also circulated extreme restrictions or unverified remedies. Caregivers wanted hospital-endorsed digital resources that were updated, readable, and tailored to children with comorbid conditions.

“Online, one article says milk causes asthma, another says milk is important. Parent groups are warm, but everyone has a different story. I need information I can trust.” (C20, father of a 10-year-old girl)

Emotional support was also part of dietary management. Caregivers wanted clinicians to recognize the labor of constant vigilance and the guilt associated with both accidental exposures and over-restriction.

“People think I am too nervous. But if something happens, they will also blame me. I want a doctor to tell the family what is necessary and what is unnecessary, so I am not alone.” (C05, mother of an 11-year-old boy)

Caregivers' suggested supports were practical but also raised implementation questions. They wanted integrated plans that could realistically be used during brief clinic visits, understood by teachers and relatives, and updated when diagnoses or asthma control changed. This suggests that future work should consider clinic workflow, dietitian availability, allergy specialist access, school health capacity, and the feasibility of maintaining written or digital action plans.

## 4 Discussion

This qualitative descriptive study explored the dietary management experiences and support needs of primary caregivers of children with coexisting asthma and food allergy. The findings should be interpreted as an in-depth account of caregivers' experiences rather than as estimates of prevalence or evidence of the effectiveness of any specific intervention. Dietary management emerged as a complex process involving risk appraisal, the establishment of protective routines, the balancing of nutritional and psychosocial needs, and coordination across home, school, clinical, and broader social contexts. Although this study directly documents caregivers' perceptions and practices, the clinical implications and potential intervention strategies discussed below represent interpretive extensions of the findings and therefore require further empirical evaluation.

The first theme, managing “double danger,” extends previous qualitative evidence that caregivers' beliefs about food can influence asthma management. Prior work among caregivers of children with asthma found that food-related perceptions could lead to dietary restriction, even when the relationship between specific foods and asthma was uncertain (14). In families with confirmed food allergy, the interpretive problem may become more complex: caregivers must avoid true allergens while resisting unnecessary restriction of foods perceived to worsen asthma. This distinction is clinically important because both under-recognition of food allergy risk and over-restriction of the diet may harm children (5, 11–13).

The culturally embedded food beliefs described by caregivers should be interpreted within the Chinese family and social context. Terms such as “heaty,” “cold,” and “phlegm-producing” helped families make sense of ambiguous symptoms and communicate risk across generations. These beliefs sometimes contributed to unnecessary restriction, but they also represented caregivers' attempts to integrate medical advice, family experience, and culturally familiar explanations. Clinicians may therefore need to ask about these categories respectfully, distinguish confirmed allergens from culturally avoided foods, and negotiate safe dietary plans without dismissing family knowledge.

These culturally mediated interpretations should also be read in relation to trust and communication. Similar qualitative work among Hong Kong Chinese parents of children with asthma has shown that caregiving distress and management choices are shaped by uncertainty, family support, and plural health beliefs (30). In the present accounts, culturally familiar food categories sometimes retained authority when professional advice was brief or fragmented. This suggests a need for future research to evaluate whether culturally responsive, diagnosis-specific explanations improve understanding without dismissing family knowledge.

The second theme, building protective food routines, is consistent with qualitative studies of pediatric food allergy showing that daily management requires label reading, preparation of safe foods, advocacy, and emergency readiness (15, 17). Comorbid asthma may intensify concern about respiratory symptoms during allergic reactions and make routine planning feel less secure. Caregivers' accounts showed that asthma action plans, food allergy emergency plans, school communication, and nutrition advice were often managed together in daily life. This points to a need for integrated counseling rather than parallel education delivered separately by different specialties.

The third theme, balancing safety, nutrition, and normal childhood, highlights the tension between necessary avoidance and the risk of excessive dietary narrowing. Avoidance diets are essential when food allergy is confirmed, but prolonged or excessive elimination may affect dietary variety, social participation, quality of life, and caregiver anxiety (12, 13, 19). Recent guidance on IgE-mediated food allergy emphasizes allergen avoidance, dietary advice, written treatment plans, education on allergic symptom recognition, appropriate medication, and psychological support when anxiety is substantial (5, 11). For children with asthma and food allergy, nutritional counseling should be framed not only as nutrient replacement but also as a strategy to reduce avoidable fear and support participation in school and family life.

Targeted literature further supports the relevance of these domains: caregiver quality of life varies with allergy profile, multiple allergies, socioeconomic context, and perceived reaction severity (31); school guidance recommends individualized action plans and staff preparation but emphasizes low certainty of evidence and local adaptation (32); and observational nutritional research links elimination-diet management and adequate substitution with growth and nutrient intake (33). These studies contextualize the caregivers' concerns but do not demonstrate that the specific strategies proposed from the present qualitative findings will improve outcomes.

The fourth theme, needing integrated, practical, and credible support, clarifies what caregivers considered useful and feasible. Families did not simply ask for more information; they wanted advice that was coherent across specialties and usable in real contexts. This interpretation aligns with previous qualitative work showing that food allergy families need professional and peer support, credible resources, mental health support, and practical solutions for daily management (15, 17). It also resonates with recent pediatric asthma qualitative research describing caregiver burden, knowledge gaps, school support needs, family disruption, and anxiety (18).

Transferability should be considered in relation to the study context. The study was conducted in a single tertiary hospital-based pediatric service in Deyang, China. Families attending such clinics may differ from families in primary care, rural areas, or regions with different access to allergy testing, dietitians, school nurses, or epinephrine. Chinese family structure may also shape dietary decisions because grandparents and extended relatives often participate in child feeding. The findings may therefore transfer most readily to settings where pediatric asthma and food allergy care is specialist-led, school food systems require caregiver negotiation, and culturally embedded food beliefs influence family decision-making.

### 4.1 Possible clinical and nutritional implications

The findings suggest several possible implications for pediatric nutrition and allergy-asthma care, while recognizing that these implications require future evaluation. First, clinicians may consider distinguishing confirmed food allergens from foods that caregivers believe may trigger asthma without adequate evidence. A structured “confirmed allergens, uncertain triggers, safe foods” discussion may help reduce both accidental exposure and unnecessary dietary narrowing. Second, a combined asthma-food allergy action plan may be useful if it clarifies symptom patterns and first steps during respiratory, skin, gastrointestinal, and systemic symptoms. Third, earlier dietitian involvement may be particularly valuable for children avoiding milk, egg, wheat, multiple protein sources, or culturally important staple foods. Fourth, schools may need concise written plans, safe meal procedures, and staff education that support inclusion in food-related activities. These approaches should be viewed as intervention-development targets rather than validated outcomes of the present study.

Accordingly, each implication above should be read as an author-inferred hypothesis grounded in caregiver accounts, not as a tested clinical recommendation. The present study did not evaluate whether integrated counseling, dietitian involvement, school letters, or combined action plans reduce exposures, dietary restriction, anxiety, or asthma morbidity; those outcomes require prospective feasibility and effectiveness studies.

Implementation may be constrained by short outpatient visits, separation between respiratory, allergy, and nutrition services, uneven access to dietitians and allergy specialists, limited availability of school health staff, variable food-labeling literacy, and inconsistent access to emergency medication. Policy and service planning may need to consider dietitian workforce capacity, school communication systems, and standardized written plans for children with overlapping asthma and food allergy risk.

### 4.2 Implications for future intervention development

Future interventions could include a multidisciplinary education session for families of children with asthma and food allergy; a one-page integrated action plan; a dietitian-led substitution and meal-planning module; school communication templates; brief videos on label reading and emergency response; and developmentally appropriate coaching for children to recognize risk, refuse unsafe foods, carry safe snacks, and communicate needs without shame. Intervention development should include caregivers, children, teachers, respiratory clinicians, allergists, dietitians, and psychologists to ensure that materials are accurate, practical, culturally acceptable, and feasible in routine care. Pilot testing should examine acceptability, usability, dietary variety, caregiver confidence, school communication, and safety outcomes before any clinical effectiveness claims are made.

Any future intervention should specify its mechanism, feasibility constraints, and measurable outcomes before benefit is inferred. The present findings identify candidate components and implementation questions only.

### 4.3 Strengths and limitations

Strengths of the study include purposive sampling across asthma control and food allergy profiles, in-depth interviews, reflexive thematic analysis, attention to clinical usability, and explicit reporting of sample characteristics and analytic procedures.

The study also has limitations. Recruitment from a single tertiary hospital may overrepresent families with more severe or complex disease, higher health literacy, or greater access to specialist services. Caregivers willing to discuss dietary management may have had stronger concerns or more developed routines than non-participants. The study relied on caregiver self-report and did not include direct interviews with children, teachers, school staff, clinicians, or dietitians. We did not objectively assess dietary intake, nutritional adequacy, growth, asthma control, actual school practices, or whether reported restrictions matched clinical advice. Recall bias and social desirability bias may have influenced accounts of food avoidance, medication use, school communication, or emergency preparedness. The analysis may not fully capture socioeconomic influences on dietary management if these data were incompletely recorded. Transferability outside the Chinese healthcare, school, and cultural context may be limited. Finally, interview mode may have influenced disclosure because some participants were interviewed by video call and others in clinic-based private rooms.

Diagnostic certainty for food allergy varied because not all diagnoses were supported by oral food challenge; sensitization testing and clinical history may not have equivalent specificity, and some misclassification is possible. The sample also included IgE-mediated, non-IgE-mediated, mixed, and unspecified phenotypes, so findings should not be generalized exclusively to IgE-mediated allergy. Asthma-control status was derived from routine clinical documentation and treating-clinician assessment rather than a study-administered standardized instrument, which may have introduced classification variability. These limitations constrain cross-case comparisons based on food-allergy phenotype or asthma-control category.

## 5 Conclusion

Primary caregivers of children with both asthma and food allergy experienced dietary management as a continuous process of risk interpretation, protective routine-building, nutritional balancing, and cross-setting coordination. The findings identify caregiver-perceived needs for support connecting asthma control, food-allergy safety, nutrition, school communication, and emotional reassurance. These insights may inform the co-development and future evaluation of family-centered nutrition and allergy-asthma support interventions.

## Data Availability

The data analyzed in this study is subject to the following licenses/restrictions: Full interview transcripts are not publicly available because they contain contextual details that could permit participant identification. De-identified excerpts and selected analytic materials may be made available by the corresponding author upon reasonable request, provided that the proposed use is consistent with participant consent, institutional policy, and additional ethics approval where required. Requests to access these datasets should be directed to Kejimu Sunzi, 819228903@qq.com.
