# Supplementary material for: Dietary management experiences and support needs of primary caregivers of children with asthma and food allergy: a qualitative descriptive study
Source: Front Nutr. 2026 Aug 12;13:1904972. doi: 10.3389/fnut.2026.1904972 (PMC13510291; doi:10.3389/fnut.2026.1904972)
Supplement: Supplementary file 1 [file Table_1.docx]

**Supplementary Table S1. Recruitment and interview flow.**

| **Item** | **Value** |
| --- | --- |
| Caregivers approached | 41 |
| Caregivers not screened | 5 (unable to contact after invitation, n = 3; no time for screening, n = 2) |
| Caregivers screened | 36 |
| Ineligible after screening | 8 (no confirmed food allergy, n = 5; child age outside 6-12 years, n = 2; therapeutic diet unrelated to food allergy, n = 1) |
| Eligible caregivers | 28 |
| Enrolled caregivers | 24 |
| Declined participation | 4 |
| Reasons for refusal | time constraints, n = 2; concern about audio recording, n = 1; topic perceived as stressful, n = 1 |
| Withdrawals after enrollment | 0 |
| Clinic-based interviews | 17 |
| Video interviews | 7 |
| Final interviews yielding no new themes | Interviews 21-24 |
