# Supplementary material for: Dietary management experiences and support needs of primary caregivers of children with asthma and food allergy: a qualitative descriptive study
Source: Front Nutr. 2026 Aug 12;13:1904972. doi: 10.3389/fnut.2026.1904972 (PMC13510291; doi:10.3389/fnut.2026.1904972)
Supplement: Supplementary file 2 [file Table_2.docx]

**Supplementary Table S2. Clinical, dietary, and caregiver-context variables reported in Table 1.**

| **Domain** | **Variables reported in Table 1** |
| --- | --- |
| Asthma | Diagnostic criteria, control tool/status, severity where available, medication use, time since diagnosis |
| Food allergy | Food allergens, diagnostic method, phenotype, reaction history, severe systemic reactions, epinephrine availability |
| Dietary management | Number/type of avoided foods, school meals/home-packed meals, nutrition assessment/growth concerns, dietitian consultation type |
| Caregiver context | Age, education, employment/occupation, socioeconomic indicator if collected, household structure |
